# Differentiating busking from begging: A psychological approach

**DOI:** 10.1371/journal.pone.0260781

**Published:** 2021-12-02

**Authors:** Robbie Ho, Wing Tung Au

**Affiliations:** 1 Division of Social Sciences, Humanities and Design, College of Professional and Continuing Education, The Hong Kong Polytechnic University, Hung Hom, Hong Kong; 2 Department of Psychology, The Chinese University of Hong Kong, Sha Tin, Hong Kong; The University of Hong Kong, HONG KONG

## Abstract

Despite the research support that street performance is generally a beneficial element to public space, the legitimacy of street performance remains controversial. One critical issue is that busking is often confused with begging. With a psychological perspective, the present research examines the distinction of busking from begging. Two studies approached the problem from the viewpoints of street performers and passersby, respectively. Study 1 (*N* = 188) surveyed street performers on their reasons for street performance and reasons for why donations to street performance should be acceptable. The respondents could articulate various features of street performance along which busking could be similar to and yet distinguishable from begging. Study 2 (*N* = 189) experimentally compared busking and begging in how they could affect people’s perception of public space. Relative to public space with begging, public space with busking was perceived as significantly more comforting, more active, less prone to crimes, and overall more likeable. These descriptive (Study 1) and experimental (Study 2) findings help to clarify the difference between busking and begging: Street performance is not merely an act of soliciting donations in public space, but it also possesses artistic and entertaining qualities that can in turn make public space more favorable. The current findings can inform the policy making and regulations of street performance. Moreover, since the present research was conducted in Hong Kong, it contributes a cultural perspective to the literature on street performance.

## Introduction

Despite the research support that street performance is generally a beneficial element to public space, the legitimacy of street performance remains challenged. One critical issue has been the confusion between busking and begging. With a psychological perspective, the present research seeks to examine the differences between busking and begging. Specifically, we conducted a survey of street performers (Study 1) and an experiment comparing busking and begging (Study 2) to understand how busking might be distinguished from begging. This paper will first provide the background to the present research. It will operationalize street performance and the related concepts, present the extant evidences for the benefits of street performance to public space, and explain the confusion between busking and begging. Since the present research happens to be conducted in Hong Kong, a brief history as well as the current regulations of the Hong Kong street performance will also be presented. Next, Studies 1 and 2 will be reported separately, including their respective methods, data analyses, and major findings. Finally, this paper will summarize the current findings and conclude the present work. Implications and suggestions for the policy making and regulations of street performance will be made. Limitations of the current studies and possible future directions will also be discussed.

## Background

### Street performance/busking

*Street performance*, or *busking*, refers to the act of performing or entertaining in public space with the intention of seeking voluntary donations from the passersby. *Street performers*, or *buskers*, are persons who conduct such an act. Throughout this paper, “street performance/street performer” and “busking/busker” are used interchangeably. “Street performance/street performer” is more familiar among American texts [1,2] whereas “busking/busker” is more familiar among British texts [3,4]. But over the years in the literature, it becomes increasingly common that the two phrases are used interchangeably [5–15]. We follow this convention in the present work.

Street performance is closely related to the concept of *public space*, which is composed of surfaces and objects necessarily owned by the city and some people [16]. Carr et al. [17] define public space as “open, publicly accessible places where people go for group or individual activities” (p. 50). Project for Public Spaces [18] defines public space as places that are “used by many different people for many different purposes at many different times of the day and the year” (p. 1). From a psychological viewpoint, experience of public space can contribute to our mental well-being. Empirical findings have shown that the perceived quality of public space is positively associated with people’s residential satisfaction [19] as well as sense of community [20]. In addition, experimental studies have found that the perception of public space can be enhanced by adding certain urban elements such as seats, sculptures, and food vendors [21,22], trees and vegetations [23,24], and street performance [25]–the subject of our interest. Reviewing various typologies of public space [17,26–28], Ho and Au [29] identify 12 major types of public space, as presented in Table 1. Street performance is a spontaneous activity, and normally no one knows where and when exactly a street performance will take place. However, among the major types of public space, street performance is mostly encountered in transitional places such as transport facilities, streets, and squares or plazas, where there are constant pedestrian foot traffics [30].

**Table 1:** 12 Major types of public space.

Type	Definition
Transport facility	Public space for transport facilities such as transit stations or stops for subways or buses
Street	Pedestrian and vehicular corridor where people move on foot
Square	Multifunctional space available to all people
Recreational space	Specialized space designed or used for sports or exercises
Found neighborhood space	Vacant or undeveloped space that is either ignored or not intended for a specific use
Park	Green area intended for social activities
Memorial	Space that memorializes people or important events
Market	Outdoor or exterior space used for shopping
Playground	Play area that includes play equipment (e.g., slides and swings)
Community open space	Space designed, developed, or managed by local residents on vacant land
Indoor marketplace	Indoor shopping area
Waterfront	Open space along waterways in cities

Street performance is occasionally associated with *political street performance*, which refers to the specific activity that utilizes performance in public space to activate and promote political agendas and discussions among the general public [4,31,32]. Some considers street performance as a gateway to empowering the general citizens the rights to public space. While any particular public space is inevitably controlled by a certain class of people, the general citizens may regain their rights to public space by supporting and participating in street performance [31]. However, our understanding of political street performance entails a conscious intention from the actor of the event. Thus, even though street performance (as well as some other activities in public space) may at times be perceived by the passersby as politically related, we do not consider it as political street performance unless its actor consciously intends to be political in the public. This implies that street performance is not necessarily related to protest or activism.

Street performance is artistic in nature. Fisher [33] defines public art as “all forms of creative expression in public space” (p. 43). Riggle [16] defines street art as “largely ephemeral art that is usually cheap to make, free to experience, and owned and overseen by no one (or rather, everyone)” (p. 249). Both these concepts of public art and street art are manifested in street performance. But unlike static artworks such as public sculptures and graffiti murals, street performance is a live performing art that entails social processes between a performer and an audience [34]. Carlin [35] describes street performances as “socially organized activities”. To negotiate and articulate their presence and the boundaries of their performances in public space, street performers must perform, engage, and interact with the passersby in real time [36]. Street performance comes in various forms, although a general differentiation can be made between musical (e.g., pop, rock, jazz, classical, etc.) and nonmusical (e.g., juggling, miming, dance, magic, etc.) busking. Particularly, musical busking, or *street music*, is a prototypical form of street performance throughout history [1–3,6]. It is also possible to classify street performance in terms of how the performer engages the spectators. Mason [4] classifies five types of street performer: *entertainer*, *animator*, *provocateur*, *communicator*, and *performing artist*. Entertainer entertains and pleases the spectators. Animator involves the spectators to perform in the show too. Provocateur challenges societal norms, conventions, and beliefs although there may not be an obvious answer to the question of interest. Communicator has a clear message and even ideology–can be religious, moral, or political–to convey to the spectators. Performing artist simply creates abstract images on the street. The art of street performance is evidenced by its variety and diversity.

Last but not least, street performance is commercial in nature. Historically, wandering minstrels, troubadours, mountebanks, and showmen in street fairs performed on the street to earn a living [3,6]. This tradition continues into the present day. Bouissac [37] describes street performance as “the modern, urban version of an ancient mode of economic survival” (p. 14). But unlike the street trades and businesses who sell their products or services at a fixed or explicitly agreed price, street performance generally does not oblige its spectators to pay. People who have watched a street performance may choose freely whether to donate money to the performer or not. Even if they choose to donate, they are free to decide how much to donate. Thus, street performance is like a public good that is open to freeriding [11]. Instead of obliging the spectators to pay, street performers typically adopt subtler approaches such as displaying signs and/or receptacles (e.g., hats, instrument cases, etc.) and/or make announcements to encourage donations [38]. Both the gives and takes in street performance are voluntary.

### The benefits of street performance to public space

Street performance has been studied from various perspectives. Plentiful attention has been paid to the history of street performance [1,3,39], individual accounts and life stories of street performers [14,40,41], and case studies of the street performances in specific cultural contexts [2,12,42]. Others have studied street performance from the economic [11], legislative [13], urban design [5,7], and spectator experience [43–45] perspectives. Our fundamental interest is in the relation of street performance with public space [8,9,15,25].

Research over the years supports the view that the presence of street performance can make public space more favorable. According to Whyte [46], street performance “provides a linkage between people and prompts strangers to talk to each other as though they were not” (p. 94). Street performance attracts and draws crowds in public space and thereby creates a sense of community [1]. In New York, train riders reported that they felt safer with street music around the subway stations [42]. In Bath, Simpson [15] observed that street performance could transform public spaces into performance places and thereby make public spaces more sociable and convivial. Similarly, in Stockholm, street music could animate and soften public space [9]. In Santa Monica, visitors to a shopping promenade thought that street performance was important to the attraction and desirability of the area [8]. In Hong Kong, audiences of street performance reported that street performance made them feel positively toward the surrounding street environments (e.g., “*This performance made me love this place*.” & “*This performance made this place feel secure*.”) [43,44]. Recently, there have been experimental data supporting the causal effect of street performance on the perception of public space. Research participants were shown images of various public spaces before and after street performance was added. On average, the participants thought that street performance could improve their perceptions of the public spaces. This was then followed up by a field experiment in Hong Kong, which found that public space with street performance was perceived more positively than public space without street performance [25]. In sum, a considerable body of research evidence is in favor of the positive impact of street performance on public space.

### Confusion between busking and begging

Despite the research support for the benefits of street performance, the legitimacy of street performance remains controversial. One major issue is the confusion between busking and *begging*. To beg means “to ask for alms” or “to ask for as a charity” [47]. Street performers do make a living by seeking donations from the passersby in public space. Thus, some people may think of busking as alike begging for that they both involve the solicitation of donations in public space. Moreover, as both busking and begging occupy public space, both may be seen as an act of vandalism or a source of nuisance. In the 1930s, New York once banned street music since buskers and beggars could not be clearly differentiated [48]. Currently, there is still no global consensus on the legitimacy of street performance; busking is legal in some places but illegal in others [10]. For example, in Australian cities such as Melbourne and Sydney, street performers may obtain licenses for performing and accepting donations legally in public space [13]. But in Hong Kong, street performers may be arrested for conducting “unauthorized charitable behavior” if they accept donations from the passersby in public space [49]. The unclear demarcation between busking and begging underlies the controversy over street performance.

However, it is debatable that busking can indeed be distinguished from begging. One important distinction lies in the artistic nature of street performance. While beggars typically offer nothing to the donor as an exchange for the donation being received, buskers consciously provide the donor with the service of performance and entertainment (and they do so before the donor donates). Moreover, buskers’ performance and entertainment have the potential to enhance the sociality and conviviality of public space [15]. In a nutshell, busking and begging may be alike for that they both solicit donations in public space, but at the same time busking should be distinguishable from begging in that it additionally provides performance and entertainment which have the potential to make public space more favorable. It is the primary goal of the present research to verify such distinction of busking from begging.

### Hong Kong as the research context

#### Brief history of Hong Kong street performance

The present research happens to be conducted in Hong Kong, and therefore some background of the Hong Kong street performance needs to be delineated. Lai and Zhou [50] provide a concise history of the Hong Kong street performance, as follows: Hong Kong street performance can be traced back to the 19^th^ century and it comprised various forms including singing, Cantonese opera, martial arts, storytelling, and animal tricks. These various forms of street performance most often took place in night markets on urban streets that were also filled with hawkers, fortune tellers, entertainers, and the likes. Street performance constituted part of Hong Kong people’s daily social and communal life until the late 20^th^ century. During the 1970s and 1980s, Hong Kong street performance declined due to a rapid urban redevelopment and the government’s scheme to clear urban streets of unauthorized activities to improve public safety and make space for vehicular traffics. However, Hong Kong street performance re-emerged in 2000 –on a large scale–when a new pedestrianization program was implemented to close off selected urban streets to ease congested pavements and improve pedestrian traffics. The program accidentally attracted and gave way to a vast variety of recreational, social, and commercial activities–street performance was one of them. In recent years, street performances have frequented in the major commercial districts in Hong Kong such as Causeway Bay, Central, and Mong Kok (pp. 6–7). Currently, the street performances in Hong Kong are dominated by musical performances of *Cantopop*. Short for Cantonese popular music, Cantopop characterizes (a) Western pop melodies and (b) modern-Chinese lyrics that are sung in Cantonese [51]. Hong Kong popular music and popular culture are strongly identified with Cantopop [52]. In other words, Cantopop is very familiar among the Hong Kong locals. In Hong Kong street performance, Cantopop is normally performed with karaoke or with the accompaniment of portable instruments such as guitars (often amplified) and acoustic percussions (e.g., tambourine and cajón). This specific music genre and presentation of Cantopop popularize and give shape to the Hong Kong street performance these days, contrasting it sharply against the Hong Kong street performance in the old days (i.e., Cantonese opera, martial arts, storytelling, and animal tricks).

#### Current regulation of Hong Kong street performance

Despite its popularity, the legality and regulation of Hong Kong street performance remain complicated. It is either that Hong Kong street performance is only fully accepted in a few restricted areas, or that its regulation in the general public space continues to be ambiguous and confusing in practice. Currently in Hong Kong, only two restricted areas legitimize street performance explicitly: Sha Tin Town Hall (STTH) [53] and West Kowloon Cultural District (WKCD) [54]. These areas are separately managed in terms of street-performance rights, although generally speaking, to perform in either area, the interested party must attend audition to obtain a permit or license beforehand. People approved and licensed for STTH are entitled to register for performing one two-hour session each on Saturdays, Sundays, and public holidays. Those approved and licensed for WKCD are entitled to perform for up to two hours within any six-hour periods between 10am and 10pm every day. Furthermore, for both areas, the licensees must perform at the exact spots that are pre-designated by the authorities–STTH has only one spot and WKCD has eight spots. Overall, although STTH and WKCD afford places where street performance can be accepted, the restrictions of who, when, and where to perform in these areas clearly limit the essence of street performance as traditionally defined, making these areas fundamentally unfriendly to street artists whose work and practice are in nature spontaneous, autonomous, and self-initiated.

With respect to the general public space other than STTH and WKCD, there is unfortunately *not* a specific government unit to oversee matters related to street performance. To date, the responsibility for managing street performance (and other street activities) is diffused among as many as nine different government departments–namely, *Environmental Protection Department*, *Hong Kong Police Force*, *Lands Department*, *Home Affairs Department*, *Food and Environmental Hygiene Department*, *Highways Department*, *Social Welfare Department*, *Transport Department*, and *Buildings Department* [49,50]. With such diffusion of responsibility, the regulation of street performance in the wider public space in Hong Kong continues to be ambiguous and confusing. Although there are no specific laws to prohibit street performance, there are at least several common laws that can limit the rights to street performance in Hong Kong, as summarized in Table 2. For instance, under the Summary Offences Ordinance [55], people are not allowed to play any musical instrument on a public street or road unless they have permission from the relevant authority. Also, people are not allowed to play any game or pastime to the annoyance of the inhabitants or passersby. In 2006, a street performer commonly known as Mr. Funny was arrested under the ordinance when he performed acrobatics on a street that was closed off for the pedestrianization program at the time [56]. The charge was dropped eventually. The judge ruled that Mr. Funny should enjoy the freedom of engaging the general public in cultural activities such as street performance [57]. But even if street performance may be permissible for its artistic and cultural qualities, street performers may still be arrested as beggars committing “unauthorized charitable activities” [49]. Also under the Summary Offences Ordinance [55], people are not allowed to wander around or place themselves in public space to beg or gather alms, or they may be liable for a fine or even imprisonment after multiple offenses. In 2012, a street musician was arrested when he played harmonica near a subway exit. He was charged for begging in public space and was fined HK$200 (approx. US$26) [57]. Lately, street performance has been banned entirely from public space one after another. In 2018, the pedestrian zone of Sai Yeung Choi Street South in Mong Kok was officially terminated [58,59]. In the same year, injunctions were granted to ban street performers from performing in the open space of Times Square in Causeway Bay [60,61].

**Table 2:** Ordinances related to the rights to street performance in Hong Kong.

Ordinance	Term no.	Details	Penalty
Cap. 228 Summary Offences Ordinance [55]	4(5)	Causes any annoyance or obstruction in any public place— (i) by exposing anything for sale in or upon, or so as to hang over, any street, road or footway, or on the outside of any house, shop or building.	Shall be liable to a fine of HK$500 or to imprisonment for 3 months.
	4(15)	Plays any musical instrument in any public street or road save under and in accordance with the conditions of any such general or special permit as the Commissioner of Police in his absolute discretion may issue.	
	4(23)	Plays at any game or pastime to the annoyance of the inhabitants or passers-by; or plays at any game or loiters in any public place, so as to obstruct the same or create a noisy assembly therein.	
	4(28)	Does any act whereby injury or obstruction, whether directly or consequentially, may accrue to a public place or to the shore of the sea, or to navigation, mooring or anchorage, transit or traffic.	
	4A	Any person who without lawful authority or excuse sets out or leaves, or causes to be set out or left, any matter or thing which obstructs, inconveniences or endangers, or may obstruct, inconvenience or endanger, any person or vehicle in a public place.	Shall be liable to a fine of HK$5,000 or to imprisonment for 3 months.
	26A	Any person who wanders abroad, or places himself or herself in any public place, street or waterway to beg or gather alms, or causes or procures or encourages any child or children so to do, commits an offence.	Shall be liable on conviction— (a) for a first or second offence, to a fine of HK$500 and to imprisonment for 1 month; (b) for a third or subsequent offence, to a fine of HK$500 and to imprisonment for 12 months.
Cap. 400 Noise Control Ordinance [62]	5(1)	Any person who at any time in any domestic premises or public place— (a) plays or operates any musical or other instrument, including any record or cassette player or radio or television apparatus; (b) uses any loud-speaker, megaphone, or other device or instrument for magnifying sound; (c) plays any game or engages in any pastime; or (d) carries on a trade or business, the noise of which is a source of annoyance to any person commits an offence.	Shall be liable to a fine of HK$10,000.

Approximately, HK$500 = US$64, HK$5,000 = US$642, and HK$10,000 = US$1,284.

To sum up, in Hong Kong, while street performance may be acceptable to the public for its artistic and cultural contributions, the authority may still charge street performers as beggars for seeking donations in public space. Currently there are no specific laws nor well-defined legal terms to clearly demarcate between busking/buskers and begging/beggars. Therefore, Hong Kong exemplifies the research problem of differentiating between busking and begging, and thus serves as a fitting context for the present investigation. In addition, majority of the extant research studies on street performance was carried out in the Western contexts [1,2,5,7–9,12,15,39,42]. Hence, researching street performance in Hong Kong will also contribute a cultural perspective to the literature.

### The present studies

With a psychological perspective, the present research aims at examining the distinction of busking from begging. We conducted two studies: Study 1 surveyed street performers on their reasons for doing street performance and reasons why they thought donations to street performance should be acceptable. Study 2 experimentally compared busking and begging in terms of their effects on people’s perception of public space.

## Study 1: Qualitative survey of street performers

### Study 1 method

#### Period and locations

We surveyed the street performers in Hong Kong over a five-week period during August and September in 2015. During this period, we carried out field study twice a week, on weekends. In each study session, we spent approximately seven hours scouting for street performers around five areas known as hotspots for street performance [50]: Tsim Sha Tsui (Star Ferry Pier and Hong Kong Cultural Centre), Mong Kok (pedestrian zone of Sai Yeung Choi Street South), Central (Central Ferry Piers and Lan Kwai Fong), Causeway Bay (Times Square and pedestrian zone of East Point Road), and West Kowloon Cultural District. Street performers whom we encountered were invited to take part in the survey. They were provided with a paper questionnaire to fill out on the spot. Some of them responded to the survey at a later time either by completing an online version or by telephone. To increase the number of survey responses, we invited the respondents to forward the survey to other street performers.

This study involving human participants was reviewed and approved by The Survey and Behavioural Research Ethics Committee of The Chinese University of Hong Kong. The respondents provided their verbal informed consent to participate in this study.

#### Respondents

Among the 188 respondents, 56 (29.8%) were women and 182 (96.8%) were Hong Kong residents. Details of the respondents are presented in Table 3. More than 80% were young adults under 29 years old. In terms of performance type, more than 90% performed music. The respondents were fairly evenly distributed in terms of (a) their number of years of busking experience ranging from “*one year or less*” to “*four years or more*”, (b) employment status outside the busking job, and (c) busking frequency ranging from “*once a month or less*” to “*twice a week or more*”.

**Table 3:** Profile of Study 1 respondents.

	*n*	%
***Age***		
19 or below	50	26.6
20 to 24	68	36.2
25 to 29	37	19.7
30 to 39	6	3.2
40 to 49	5	2.7
50 to 59	15	8.0
60 or above	5	2.7
Preferred not to answer	2	1.1
***Performance type*** *		
Music	177	94.1
Dance	14	7.4
Acrobatics	4	2.1
Theater	2	1.1
***Busking experience***		
One year or less	54	28.7
Two years	47	25.0
Three years	43	22.9
Four years or more	42	22.3
Preferred not to answer	2	1.1
***Employment status outside busking***		
Full-time job	71	37.8
Part-time job	64	34.0
No other job	47	25.0
Preferred not to answer	6	3.2
***Busking frequency***		
Once a month or less	38	20.2
Once a month to twice a month	26	13.8
Twice a month to once a week	58	30.9
Once a week to twice a week	45	23.9
Twice a week or more	20	10.6
Preferred not to answer	1	0.5

* More than one choice might be selected.

#### Questions

The respondents were asked two open-ended questions. The first question probed their reasons for street performance (“*Why do you do street performance*?”). The second question probed why they thought donations to street performance should be acceptable (“*Why do you think that donation to street performance is acceptable*?”). The respondents were reminded to answer as openly and fully as they reasonably could, and that there were no right or wrong answers.

### Study 1 data analysis and findings

We analyzed the collected data to identify the unique responses to each of the two open-ended questions. We coded the respondents’ answers to each question. Results of coding, with selected excerpts, are presented in Tables 4 and 5. For the first question on the street performers’ reasons for street performance, 241 responses were coded and they could be summarized into eight unique themes (Table 4): (i) *interest and self-entertainment*, (ii) *to share and promote arts and culture*, (iii) *learning and experiences*, (iv) *to provide entertainment*, (v) *income*, (vi) *to enhance sociality and conviviality of the city*, (vii) *self-promotion*, and (viii) *to demonstrate rights to public space*. For the second question on the reasons why donation to street performance should be acceptable, 228 responses were coded and they could be summarized into five unique themes (Table 5): (i) *street performance is work*, (ii) *support for street performers*, (iii) *donation is voluntary*, (iv) *support for street performance culture*, and (v) *donation is natural*.

**Table 4:** Unique themes of the reasons for street performance.

Theme (number of responses)	Selected excerpts
(i) Interest and self-entertainment (104)	• Because I like singing and playing guitar.
	• I want to go out to sing some songs, as karaoke can no longer satisfy my desire for singing!
	• I like playing drums.
	• I like to be with my friends to do what we love doing, and I simply like being able to dance in any environment at any time.
(ii) To share and promote arts and culture (57)	• I would like to promote the culture and concept of street performance.
	• Street art may breed many different kinds of art.
	• To do music education.
	• To share stories.
	• I want to promote more different types of music to Hong Kong.
	• To promote traditions and the quintessence of Chinese culture.
	• To promote freestyle football.
	• I want to promote poi performance in Hong Kong.
(iii) Learning and experiences (24)	• To practice one’s performance skills.
	• Because it’s too loud for the neighbor, I need to go outside for practice.
	• Because I want to increase my self-confidence through being understood by the audience and appreciated by others.
	• Since we perform on the street spontaneously without fixed time and fixed location, majority of the audience we encounter are people who hear our music for the first time. If they are willing to stay or even tell us they are moved by our music, such sense of success is priceless.
(iv) To provide entertainment (14)	• To provide a sense of harmony through music to the audience and to interact with citizens.
	• To bring positive energy.
	• Music can help people with their emotions and provide free entertainment to citizens.
	• Seeing children dancing to our music, I was “super” moved.
(v) Income (14)	• For livelihood.
	• Short of money.
	• Don’t want to get a job.
	• Since retirement I have had no income and started street performance.
(vi) To enhance sociality and conviviality of the city (13)	• Performance allows people to have a meeting place in an indifferent city.
	• Hong Kong people have begun to not find any reason for going out; I hope that people will go out to watch busking.
	• I think busking has brightened up the whole city and that makes our city more vivid and joyful.
	• This concrete jungle of Hong Kong is in great need for spirituality and spiritual food.
(vii) Self-promotion (10)	• I love singing, I want more people to know my voice.
	• I want many people to recognize and know about my music.
	• To get more performance opportunities.
	• To promote my team to realize our dream.
(viii) To demonstrate rights to public space (5)	• To exercise basic human rights.
	• To open up public space.
	• To fight for the rights and interests of public space through busking.
	• I hope that there will be legal street performances in Hong Kong one day.

**Table 5:** Unique themes of the reasons why donation should be acceptable.

Theme (number of responses)	Selected excerpts
(i) Street performance is work (63)	• In my view, street performer is no different from other professions, and the donation from passersby is like salary.
	• In fact, performance is the same as going to work: You have to contribute first, get the boss’s approval, and then receive the salary.
	• In most developed countries, street performer is a formal profession.
	• Street performance is hard-earned money, it is work.
	• Street performers need to make a living, and equipment and time etc. are costs.
	• Both practice and purchase of musical instruments are costs; donation may offset part of those costs.
(ii) Support for street performers (59)	• Behind a performance, the performer’s effort or dedication–be it magician, dancer, etc.–is the product of spending a certain amount of time practicing, can be several years or more than ten years.
	• Self-sufficiency is appreciated by the passersby.
	• The spectators should support their favorite artists.
	• Buskers make good use of public space, they are hoping for recognition and appreciation.
(iii) Donation is voluntary (44)	• Donation is not mandatory; every passerby has his/her own choice.
	• Donation is the passersby’s voluntary act.
	• People who stop to watch a street performance would assess the performer’s standard and the performance’s quality to offer a certain amount of money as a price for watching the performance.
	• To let everyone experience autonomous choice.
(iv) Support for street performance culture (39)	• This is just a kind of support for street music.
	• It shows encouragement to the musicians, allowing them to continue to add artistic vibe to the city.
	• Busking is healthy for a city’s culture.
(v) Donation is natural (23)	• In essence, busking allows donation.
	• The whole world is like this: singing, tipping, cultural exchange.
	• I disagree with the ideology that “art without asking for money seems to be greater”.

As presented in Table 6, the 8 + 5 = 13 unique themes were coded either as a feature that could distinguish busking from begging or not. The unique themes generated with our sample of Hong Kong street performers were largely aligned with our expectation about how busking could be similar to and yet distinctive from begging. On the one hand, the respondents discussed the necessary commercial nature–the donation feature–of street performance, along which busking could be similar to begging. From the respondents’ viewpoint, income was one of the reasons for street performance, and the voluntary donations from the passersby in public space naturally provided the income they were seeking. This is similar to the aim of begging, which is to gather alms and solicit donations from the passersby in public space. On the other hand, the respondents described various features of street performance along which busking could be distinguished from begging. Those features mostly reflected the artistic nature of street performance. The respondents believed that through street performance they could provide entertainment to the general public, share and promote arts and culture, and help to enhance the sociality and conviviality of public space. The respondents also took pride in street performance. Through street performance they could gain feelings of pleasure, new experiences, and opportunities for self-promotion and other performance jobs. Furthermore, the respondents treated street performance as demanding work that required them to put effort into presenting their arts and crafts on the street to gain recognition and appreciation from the general public. Overall, these features, as recognized and articulated by the street performers themselves, provide support for the distinction of busking from begging. One unique theme did not fit into neither of the aforementioned categories. For some respondents, one of the reasons for street performance was to demonstrate their rights to public space. While this is aligned with the association of street performance with political street performance, we believe that the political rights to public space are not restricted to the exercise of street performance.

**Table 6:** Unique themes as features distinguishing busking from begging.

Unique themes	Distinct from begging
Income	No
Donation is voluntary	No
Donation is natural	No
Street performance is work	Yes
Support for street performers	Yes
Support for street performance culture	Yes
To provide entertainment	Yes
To share and promote arts and culture	Yes
To enhance sociality and conviviality of the city	Yes
Interest and self-entertainment	Yes
Learning and experiences	Yes
Self-promotion	Yes
To demonstrate rights to public space	Not applicable

## Study 2: Experiment comparing busking and begging

Study 2 experimentally compared how busking and begging affect people’s perception of public space. On the premise that street performance is a beneficial element to public space [8,9,15,25], the presence of busking should yield a more favorable overall perception of public space than the presence of begging. Hence, we hypothesize that:

*Relative to public space with begging*, *public space with busking will be perceived as more likeable*. (H1)

We also compared how busking and begging could affect some particular attributes in the perception of public space. Ho and Au [29] identify eight attributes in the perception of public space: *comfort*, *activity*, *legibility*, *enclosure*, *complexity*, *crime potential*, *wildlife*, and *lighting*. Comfort is the extent to which a public space is perceived as pleasant and relaxing. Activity is the extent to which a public space is perceived as interesting and lively. Legibility is the extent to which a public space is perceived as easy to navigate within. Enclosure is the extent to which a public space makes its viewers feel enclosed. Complexity refers to how much is going on in a public space. Crime potential refers to how much a public space is perceived as prone to crimes. Wildlife refers to the amount of trees, plants, and potential wildlife in a public space. Lighting refers to the brightness and lighting quality of a public space. Among these eight attributes, we theorize that busking and begging should differ in how they affect the comfort, activity, and crime potential of public space. Unlike begging, busking is supposed to provide entertainment and should thereby make public space appear more pleasant and more interesting. Hence, we hypothesize that:

*Relative to public space with begging*, *public space with busking will be perceived as more comforting*. (H2)

*Relative to public space with begging*, *public space with busking will be perceived as more active*. (H3)

In addition, although both busking and begging involve the solicitation of donations in public space, the donation in busking can be justified by the service of entertainment that street performers provide. Thus, busking should be seen as less threatening or less associated with criminal acts than begging. Hence, we hypothesize that:

*Relative to public space with begging*, *public space with busking will be perceived as less prone to crimes*. (H4)

### Study 2 method

#### Design

We employed a one-way between-subjects design to compare the perceptions of three conditions of public space: public space with busking (busking), public space with begging (begging), and public space without busking or begging (control). Across these three conditions, the public space should be identical and we would only manipulate the presence of busking or begging.

#### Participants

College students in Hong Kong were recruited to participate in the study. They were psychology undergraduates and they received course credit as an incentive for participation. A total of 189 participants were recruited. The sample comprised 121 women and 67 men (1 participant preferred not to answer). The mean age was 18.8 years old (*SD* = 1.2; 10 participants preferred not to answer). The participants were randomly assigned to the busking (*n* = 64, 33.9%), begging (*n* = 64, 33.9%), and control (*n* = 61, 32.3%) conditions.

This study involving human participants was reviewed and approved by The Survey and Behavioural Research Ethics Committee of The Chinese University of Hong Kong. The participants provided their written informed consent to participate in this study.

#### Stimuli

Static images were used to simulate the three conditions of public space. Static images are widely adopted to assess the perception of public space [21–25,29,63,64]. Also, a meta-analysis of 84 empirical findings shows that evaluations of environments obtained via viewing static images are strongly correlated with those obtained onsite at *r* = .86 [65]. Therefore, static images should be a valid means for simulating the experience of public space.

**Fig 1:**
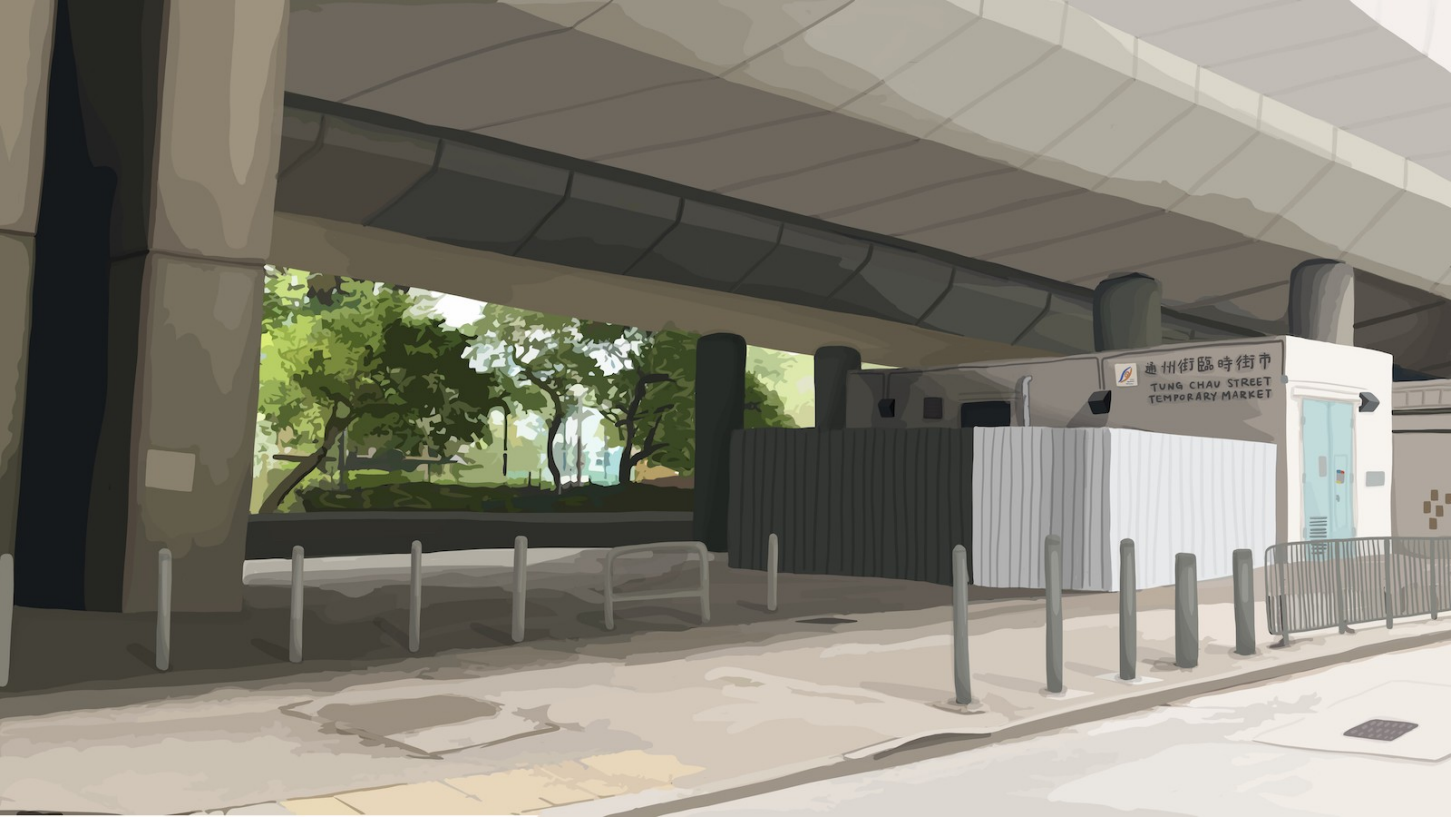
Control image. Public space without busking or begging. Image republished from Ho, Robbie, and Au, Wing Tung. “Scale Development for Environmental Perception of Public Space.” Frontiers, Frontiers, 1 Jan. 1AD, https://www.frontiersin.org/articles/10.3389/fpsyg.2020.596790/full. under CC-BY license.

**Fig 2:**
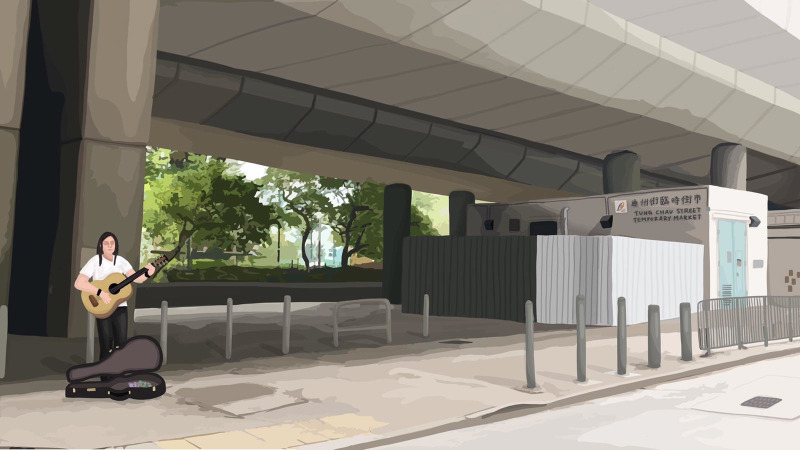
Busking image. Public space with busking.

**Fig 3:**
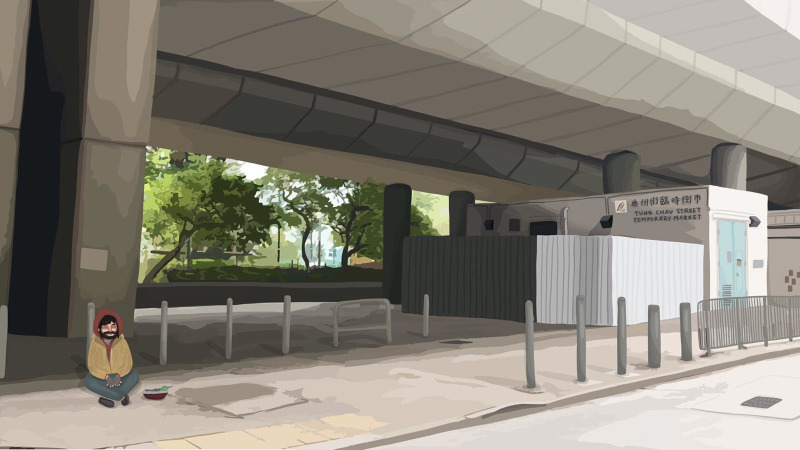
Begging image. Public space with begging.

Figs 1–3 are the three images representing the respective conditions of public space. The control image (Fig 1) belonged to a larger collection of images created by Ho and Au [29] to depict some actual public spaces in Hong Kong. The control image portrayed an empty street space suitable for pedestrian foot traffic. Based on this control image, images for the busking (Fig 2) and begging (Fig 3) conditions were created. For the busking condition, a busker was superimposed on the control image. The busker was portrayed as performing and playing an acoustic guitar; an open guitar case was placed at the front of the busker and depicted as a receptacle for donations. For the begging condition, a beggar was superimposed on the control image. The beggar was portrayed as doing nothing and sitting on the ground; a receptacle for donations was also placed at the front of the beggar. The busker and the beggar were superimposed at the same spot in the control image. This design of busker vs. beggar images was based on our initial theorization as well as the survey findings of Study 1 regarding how busking and begging should contrast each other–i.e., while busking and begging are similar in that they both solicit donations in public space, buskers typically provide performance and entertainment as an exchange whereas beggars typically offer nothing in return.

#### Measurements

All scale items are presented in Table 7. Items for measuring the overall liking of public space were adopted from Ho and Au’s [25] experiment on the impact of street performance on public space. These items were relevant to examining H1. Items for measuring the eight attributes of the perception of public space (i.e., comfort, activity, legibility, enclosure, complexity, crime potential, wildlife, and lighting) were adopted from Ho and Au [29]. This particular item set was devised and validated to capture the comprehensive perception of public space. Among the items, only those measuring comfort, activity, and crime potential were relevant to our hypotheses–H2, H3, and H4. Other items measuring constructs such as wildlife and lighting should be irrelevant to differentiating busking and begging. However, instead of including only what we expected to be relevant to our hypotheses, we decided to include the entire item set to (a) respect the original comprehensive view of public space and (b) explore if busking vs. begging would affect aspects of public space where they were not expected to do so. Comfort and activity items were rated in a 7-point bipolar format, and all the other items were rated in a 7-point Likert scale (from *strongly disagree* to *strongly agree* coded from 1 to 7 with the midpoint *neither agree nor disagree* as 4).

**Table 7:** Scale items for Study 2.

***Comfort***
Distressing–Relaxing
Fearful–Safe
Uncomfortable–Comfortable
Upsetting–Calming
***Activity***
Dull–Lively
Inactive–Active
Sleepy–Arousing
Unstimulating–Stimulating
***Legibility***
In this place it would be very easy to figure out where I am at any given moment.
In this place it would be very easy to find my way around.
In this place it would be very easy to find out my way back to any given point.
It is very easy to structure and organize this place as a picture.
***Enclosure***
In this place I strongly feel being “inside looking out”.
This place gives me a strong feeling of being enclosed in a hiding place.
This place is very cramped.
This place is very stuffy.
***Complexity***
A great deal is going on in this place.
There is a lot to look at in this place.
This place contains many elements of different kinds.
To a large extent this place promises more to be seen if I could walk deeper in it.
***Crime potential***
There are many areas in this place where a potential criminal can hide.
There is a large probability that an ill-intentioned person would hide in this place.
There is possible danger from other people in this place.
This place is prone to crimes.
***Wildlife***
In this place, there is some wildlife that can harm people, such as snakes, bees, and toxic plants.
There are many trees, vegetations, and flowers in this place.
There are potentially harmful animals and plants in this place.
***Lighting***
The light in this place is very good.
This place has uniform lighting.
This setting has very bright, clear lighting.
***Overall liking***
I like this place a great deal.
I like this place very much.
I would enjoy this place a lot.
I would really enjoy this place.

#### Procedure

Data were collected through an online experiment. The research participants were presented with the image of public space corresponding to the condition they had been assigned to. The participants were prompted to imagine that they encountered the public space on a regular basis (“*Imagine that you use this place or commute through it on a regular basis; that is*, *you encounter this place for your everyday activities*, *e*.*g*., *walking through this place to work or school*, *hanging out*, *meeting people*, *shopping*, *etc*.”). They were then asked to evaluate their perception of the public space being portrayed. They were reminded to focus on the public space rather than the quality of the image, and that there were no right or wrong answers.

### Study 2 data analysis and findings

#### Mean scores

Simple unit weighting was used to compute the composite scores for all variables that we measured. Table 8 reports the Cronbach’s alphas of and correlations among the variables. Scales that measured enclosure and wildlife had low reliability. The Cronbach’s alpha of enclosure was .46 and it could not be improved. The Cronbach’s alpha of wildlife was .39 and it was improved to .72 by removing one of the items (“*There are many trees*, *vegetations*, *and flowers in this place*”). Otherwise, the Cronbach’s alphas of all scales ranged from .69 to .91, indicating acceptable-to-excellent reliability of the measurements. Table 9 summarizes the mean scores of the three conditions of public space.

**Table 8:** Correlations and Cronbach’s alphas in Study 2.

		1	2	3	4	5	6	7	8	9
1	Comfort	.*83*								
2	Activity	.43*	.*80*							
3	Legibility	.48*	.31*	.*74*						
4	Enclosure	-.29*	-.09	-.20**	.*46*					
5	Complexity	.39*	.36*	.37*	.13	.*69*				
6	Crime potential	-.41*	-.15**	-.24**	.29*	-.12	.*78*			
7	Wildlife	-.21**	-.01	-.04	.16**	.11	.23**	.*72*		
8	Lighting	.42*	.37*	.39*	-.16**	.39*	-.31*	.04	.*73*	
9	Overall liking	.57*	.45*	.49*	-.11	.57*	-.31*	.06	.59*	.*91*

* *p* < .001

** *p* < .05; Cronbach’s alphas are presented along the diagonal.

**Table 9:** Mean scores in Study 2.

	Busking		Begging		Control	
	*M*	*SD*	*M*	*SD*	*M*	*SD*
Comfort	4.45	0.95	3.77	1.13	3.90	1.02
Activity	3.75	1.08	3.18	0.90	2.87	0.88
Legibility	4.03	0.89	3.84	0.98	3.82	1.02
Enclosure	3.98	0.71	4.10	0.75	3.84	0.82
Complexity	3.47	1.06	3.60	0.88	3.31	0.93
Crime potential	4.14	0.98	4.58	0.95	4.27	0.96
Wildlife	3.11	1.15	3.11	1.22	2.99	1.12
Lighting	3.34	1.08	3.16	1.01	3.17	1.15
Overall liking	3.30	1.09	2.87	1.00	2.71	1.14

*M* = mean, *SD* = standard deviation.

#### Hypothesis testing

We examined the mean scores among the three conditions of public space by two a priori contrasts comparing (a) busking vs. control (C1: busking = 1, begging = 0, control = -1) and (b) busking vs. begging (C2: busking = 1, begging = -1, control = 0). Complete results are presented in Table 10.

**Table 10:** Contrasts of Busking vs. Control and Busking vs. Begging in Study 2.

**Busking vs. Control**				
	Mean difference	Standard error	*t*(186)	*p*
Comfort	0.55	0.19	2.96	.003
Activity	0.88	0.17	5.16	.000
Legibility	0.20	0.17	1.18	.241
Enclosure	0.13	0.14	0.98	.330
Complexity	0.16	0.17	0.94	.348
Crime potential	-0.13	0.17	-0.78	.438
Wildlife	0.12	0.21	0.56	.573
Lighting	0.16	0.19	0.85	.399
Overall liking	0.59	0.19	3.04	.003
**Busking vs. Begging**				
	Mean difference	Standard error	*t*(186)	*p*
Comfort	0.68	0.18	3.74	.000
Activity	0.57	0.17	3.38	.001
Legibility	0.18	0.17	1.08	.284
Enclosure	-0.12	0.13	-0.90	.367
Complexity	-0.13	0.17	-0.78	.434
Crime potential	-0.44	0.17	-2.59	.010
Wildlife	0.00	0.21	0.00	1.000
Lighting	0.18	0.19	0.93	.356
Overall liking	0.43	0.19	2.25	.026

*t* = *t* value, *p* = *p* value.

Relative to public space without busking, public space with busking was perceived as significantly more comforting (*M*s = 4.45 vs. 3.90, *t*(186) = 2.96, *p* = .003), more active (*M*s = 3.75 vs. 2.87, *t*(186) = 5.16, *p* < .001), and overall more likeable (*M*s = 3.30 vs. 2.71, *t*(186) = 3.04, *p* = .003). No significant differences were found in legibility, enclosure, crime potential, complexity, wildlife, and lighting.

Relative to public space with begging, public space with busking was perceived as significantly more comforting (*M*s = 4.45 vs. 3.77, *t*(186) = 3.74, *p* < .001), more active (*M*s = 3.75 vs. 3.18, *t*(186) = 3.38, *p* = .001), less prone to crimes (*M*s = 4.14 vs. 4.58, *t*(186) = -2.59, *p* = .010), and overall more likeable (*M*s = 3.30 vs. 2.87, *t*(186) = 2.25, *p* = .026). Thus, H1, H2, H3, and H4 were all supported. No significant differences were found in legibility, enclosure, complexity, wildlife, and lighting.

#### Mediation analysis

We performed mediation analyses to explore the processes underlying the differences between busking and begging. Two dummy variables, *X*1 and *X*2, were created to code the three conditions of public space. Busking was treated as the reference group (*X*1 = 0, *X*2 = 0). *X*1 stood for control relative to busking (*X*1 = 1, *X*2 = 0). *X*2 stood for begging relative to busking (*X*1 = 0, *X*2 = 1). Using PROCESS [66] with 5,000 bootstrap samples, we tested each of the eight attributes of public-space perception, separately, to determine if they could mediate the relative effects of *X*1 and *X*2 on the overall liking of public space. We did not conduct one single mediation analysis including all eight potential mediators together out of concern for their multicollinearity–i.e., they were moderately correlated with each other (Table 8).

Complete results are presented in Table 11. All models significantly predicted overall liking of public space (all *p*s < .05). Here we describe selectively the significant indirect effects. Comfort mediated the relative effects of control vs. busking (*X*1: *b* = -0.32, *SE* = 0.10, 95% CIs [-0.52, -0.12]) and begging vs. busking (*X*2: *b* = -0.39, *SE* = 0.12, 95% CIs [-0.65, -0.17]). Thus, relative to both control and begging, busking enhanced the perception of comfort, which subsequently led to more liking. Activity also mediated the relative effects of control vs. busking (*X*1: *b* = -0.40, *SE* = 0.11, 95% CIs [-0.63, -0.20]) and begging vs. busking (*X*2: *b* = -0.26, *SE* = 0.09, 95% CIs [-0.46, -0.10]). Thus, relative to both control and begging, busking enhanced the perception of activity, which subsequently led to more liking. Finally, crime potential mediated the relative effect of begging vs. busking (*X*2: *b* = -0.15, *SE* = 0.07, 95% CIs [-0.32, -0.03]). Thus, relative to begging, busking lessened the perception of crime potential, which subsequently led to more liking. These results show that comfort, activity, and crime potential could explain why public space with busking was perceived as more likeable than public spaces without busking and with begging.

**Table 11:** Mediation analysis of the relative effects of Control/Begging vs. Busking on overall liking in Study 2.

Model statistics with mediator…					Direct effects				Indirect effects			
	*F*(3, 185)	*p*	*R* ^2^		*b*	*SE*	*t*	*p*	*b*	*SE*	*LLCI*	*ULCI*
Comfort	31.70	.000	0.34	*X*1	-0.27	0.17	-1.65	.102	-0.32	0.10	-0.52	-0.12
				*X*2	-0.04	0.17	-0.22	.829	-0.39	0.12	-0.65	-0.17
Activity	15.95	.000	0.21	*X*1	-0.19	0.19	-0.98	.330	-0.40	0.11	-0.63	-0.20
				*X*2	-0.17	0.18	-0.94	.350	-0.26	0.09	-0.46	-0.10
Legibility	22.60	.000	0.27	*X*1	-0.48	0.17	-2.80	.006	-0.11	0.09	-0.29	0.08
				*X*2	-0.33	0.17	-1.96	.051	-0.10	0.09	-0.28	0.08
Enclosure	4.31	.006	0.07	*X*1	-0.61	0.19	-3.17	.002	0.02	0.03	-0.03	0.09
				*X*2	-0.41	0.19	-2.14	.033	-0.02	0.03	-0.09	0.03
Complexity	36.20	.000	0.37	*X*1	-0.48	0.16	-3.05	.003	-0.11	0.12	-0.34	0.12
				*X*2	-0.52	0.16	-3.30	.001	0.09	0.11	-0.14	0.31
Crime potential	9.62	.000	0.14	*X*1	-0.54	0.19	-2.93	.004	-0.04	0.06	-0.16	0.08
				*X*2	-0.28	0.19	-1.52	.131	-0.15	0.07	-0.32	-0.03
Wildlife	3.46	.018	0.05	*X*1	-0.58	0.19	-3.01	.003	-0.01	0.02	-0.06	0.03
				*X*2	-0.43	0.19	-2.25	.026	0.00	0.02	-0.04	0.04
Lighting	37.97	.000	0.38	*X*1	-0.49	0.16	-3.13	.002	-0.10	0.12	-0.33	0.14
				*X*2	-0.33	0.16	-2.10	.037	-0.10	0.11	-0.33	0.11

*F* = *F* value, *p* = *p* value, *b* = unstandardized coefficient, *SE* = standard error, *t* = *t* value, *LLCI* = lower level for confidence interval, *ULCI* = upper level for confidence interval, *X*1 = control relative to busking, *X*2 = begging relative to busking.

## Discussion

### Summary of the present studies

Street performance is supposed to be a unique activity in public space that possesses both artistic and commercial features. As they perform their arts and crafts in public space, street performers expect to receive donations from the passersby for a living. Despite the extant research support that street performance is a beneficial element to public space, the legitimacy of street performance remains challenged. One critical issue is the confusion between busking and begging. In response to that, we conducted two studies in Hong Kong to assess the distinction of busking from begging through the viewpoints of street performers and passersby, respectively.

Study 1 surveyed the street performers in Hong Kong on their reasons for street performance and reasons why they thought donations to street performance should be acceptable. The respondents’ answers were aligned with our expectations about how busking should be similar to and yet distinguishable from begging. On the one hand, busking and begging could be alike given their common donation feature. On the other hand, busking could be distinguished from begging because begging lacked artistic features. Through street performance, the street performers intended to provide entertainment, share and promote arts and culture, and help to enhance the sociality and conviviality of public space. Street performers treated street performance as hard work that required them to put effort into performing in public space to gain recognition from the passersby. Furthermore, the street performers took pride in street performance in that they felt they could gain pleasure, experiences, and opportunities from performing on the street. These qualities are obviously unique to busking and they are *not* found in begging. In other words, from the street performers’ viewpoint, busking is not merely an act of soliciting money on the street as in begging.

Study 2 experimentally compared busking against begging in terms of how they could affect people’s perception of public space. Generally, the public space with busking was perceived more favorably than the public space with begging, supporting our research hypotheses. In addition, although we measured the entire original item set of perception of public space [29], significant effects were only found in comfort, activity, and crime potential but not the rest. Similarly, only comfort, activity, and crime potential were significant in mediating the relative effect between busking and begging on the overall liking of public space. This highlights the psychological mechanisms over which busking and begging differ–relative to the presence of begging, the presence of busking could make public space appear more comforting, more active, less prone to crimes, and subsequently, more likeable. Finally, the public space with busking was also perceived more favorably than the public space without busking. In other words, the presence of street performance could cause improvement in people’s perception of public space, confirming previous research findings regarding the positive impact of street performance on public space. This provides further support regarding the distinction between busking and begging.

Findings of Studies 1 and 2 converge on the conclusion that, despite busking and begging are in common for their donation features, busking should be considered as a unique activity distinguishable from begging for its performance and entertainment values that the current studies have shown to enhance the perception of public space. Put together, the present research contributes to the literature by clarifying the distinction of street performance through the viewpoints of the street performers and the passersby, respectively, and it does so in the Asian context of Hong Kong, adding a cultural perspective to the literature on street performance.

### Practical implications

The current findings have some practical implications. Legitimacy of street performance essentially depends on the desirability of street performance to public space [5]. If street performance is seen as undesirable, then it makes sense to impose restrictions to limit its negative impact on public space. But if street performance is seen as desirable, then it is only reasonable to promote it among the general public through constructive policy and regulation terms. The desirability and hence, legitimacy, of street performance has been disadvantaged by the confusion between busking and begging. Clarifying such confusion is critical for the effective management of street performance. The current findings suggest that street performance is a unique activity that is not to be confused as the act of merely gathering alms. Future policy making and regulations should reflect this distinction. In our opinion, it can be a worthwhile strategy, broadly speaking, to legitimize explicitly the donation aspect of street performance. Authorities and policy makers need to recognize that, a freer environment for street performers to operate may lead to a better culture of street performance in the long run. One of our major findings is that busking is distinct (from begging) in terms of its performance and entertainment values. Logically, street performance should be seen less as a mere act of gathering alms as its quality increases. Apart from the street performers’ self-motivation and self-discipline, we believe that donation provides a practical incentive for street performers to improve and perfect their performance quality. The amount of donation, in theory, should inform street performers about their performance quality. But legitimate donation is necessary for this operation to take place in reality. Under restriction and arrestment of donation-seeking behaviors, street performers are deprived of the opportunity to be informed about their performance quality. Both their potential quality and income are capped at a short ceiling. Therefore, legitimizing donation should allow the general public to convey to the street performers about their performance quality via donation, and a freer environment as such will provide street performers with a real incentive to improve their practices. In the long run, that is a key to cultivating a better culture of street performance. Certainly, further research may be conducted to verify the link between performance quality and donation, which is the very basis on which the current opinion is built. Follow-up interviews with street performers may be conducted to determine the extent to which donation is indeed an incentive for them to polish their skills and qualities. Other non-monetary incentives for better performance quality may also be probed. Beyond the street performers’ own viewpoint, field studies involving observation and/or field experiment may be considered to more systematically clarify the role of donation in street performance.

### Limitations

The present research is limited to the context of Hong Kong, and so the current findings may not generalize to other places or cultures. People from different places and cultures may perceive street performance differently. Street performance may be more common in some places but rarer in others. We are not sure if the commonness of street performance in a given culture can affect how people perceive street performance. The same applies to the perception of begging. Begging may be more or less common in different places and cultures. Depending on the cultural context, some people may see begging as a mundane activity whereas other people may see it as a threatening act in public space. That may affect how begging is perceived differently from busking. Street performance is a fairly common phenomenon in Hong Kong and is known to be controversial as evidenced in the cases of arrest of street performers. It is against this cultural backdrop the current findings were obtained. Interpretation of the current findings should be taken with the Hong Kong’s cultural factors borne in mind. Future research may consider a cross-cultural approach to examine if the distinction between busking and begging can be moderated by cultural factors.

The present investigation might also be limited by the relatively young age of the current samples. The respondents in Study 1 were mainly young adults below 29 years old. The participants in Study 2 were solely college students. It is possible there were underlying properties among young adults that could have biased the current findings. Perhaps young people tend to perceive street performance more positively than do older people. We are not sure if samples of different–e.g., older–age groups would yield different responses regarding the distinction between busking and begging. Generalization of the current findings to the wider population should be taken with caution. Future studies should consider employing samples of more diverse demographic profiles.

The current findings are limited to musical busking and may not generalize to other forms of street performance such as nonmusical busking. In Study 1, over 90% of the respondents were musical buskers. In Study 2, we presented street performance as a musical busker. Theoretically–and empirically as evidenced in the current studies–the key distinction of busking from begging lies in the artistic and entertainment features of street performance. As long as a street performance demonstrates its artistic and entertainment features, it should not be mistaken as begging. Our interpretation is that, the current findings should generalize to nonmusical busking. Further studies incorporating nonmusical busking can validate this speculation.

There is the limitation that the findings of Study 2 are only based on one type of public space. As mentioned in the beginning, public space encompasses a vast variety of places with different functions and purposes. There are at least 12 common types of public space (Table 1) [29]. Since the current study only adopted one public space type (an empty street space) in comparing busking and begging, some crucial factors affecting both the perceptions of busking and begging could have been overlooked. For example, the difference between busking and begging could have been minimal or even nonsignificant had we compared them in a transport facility. Conversely, perhaps the difference between busking and begging could have been more prominent in a park or along a waterfront. Or perhaps both busking and begging would be detrimental to the perception of a memorial. In other words, comparison between busking and begging might vary as a function of public space type. In defense, the public space selected for the current study is a major representative that is typically encountered in most people’s daily life, and so the current findings should have merit in helping us differentiate busking and begging in terms of their effects on perceiving public space in general. Future studies may consider extending the busking-begging comparison to other major types of public space.

Last but not least, the findings of Study 2 may be limited by the sole use of static images in simulating the various conditions of public space. Perceiving environments in reality and via static images yield different experiences of the environments. In reality, environments are experienced and perceived through multiple modalities (e.g., vision, hearing, smell, etc.). Via images, environments are experienced and perceived through only a single modality (i.e., vision only). In addition, while experience of environment in reality is immersive, an image of an environment only provides a restricted viewpoint into the environment. The findings of Study 2 were largely based on the simulation of public space via static images and that may limit the generalizability of the findings to the situations in real life. However, static images afforded us stronger experimental control over the research participants’ experience of the public spaces with and without busking or begging. We were able to standardize the presentation of public space across the experimental conditions. Future studies may adopt stimuli more approximate our real-life experiences of public space. Video and virtual reality should better capture the motion of an environment. Image accompanied with audio is also an alternative. On-site evaluation, while affording less experimental control, can enhance the ecological validity of research findings.

## Supporting information

S1 File Experimental data for Study 2. (SAV)
